# C-Reactive Protein, Neutrophil-to-Lymphocyte Ratio, and Long-Term Mortality in Chinese Centenarians

**DOI:** 10.1001/jamanetworkopen.2023.40307

**Published:** 2023-10-30

**Authors:** Qiao Zhu, Qian Zhang, Chen Chen, Miao Liu, Yao He, Yali Zhao, Shihui Fu

**Affiliations:** 1 Central Laboratory, Hainan Hospital of Chinese People’s Liberation Army General Hospital, Sanya, China; 2 Department of Neurology, Hainan Hospital of Chinese People’s Liberation Army General Hospital, Sanya, China; 3 Department of Burns and Plastic Surgery, Hainan Hospital of Chinese People’s Liberation Army General Hospital, Sanya, China; 4 Department of Statistics and Epidemiology, Graduate School, Chinese People’s Liberation Army General Hospital, Beijing, China; 5 Institute of Geriatrics, Beijing Key Laboratory of Research on Aging and Related Diseases, National Clinical Research Center for Geriatrics Diseases, Chinese People’s Liberation Army General Hospital, Beijing, China; 6 Department of Cardiology, Hainan Hospital of Chinese People’s Liberation Army General Hospital, Sanya, China; 7 Department of Geriatric Cardiology, Chinese People’s Liberation Army General Hospital, Beijing, China

## Abstract

This cohort study explores whether biomarkers of inflammation-related aging, including C-reactive protein (CRP) and neutrophil-to-lymphocyte ratio, are associated with long-term mortality in Chinese centenarians.

## Introduction

Epidemiological studies have shown that inflammation is a factor associated with of morbidity and mortality in the elderly. The term *inflammaging* has been proposed to describe inflammation that develops with age and is associated with susceptibility to aging-related pathologies.^1^ C-reactive protein (CRP) and neutrophil-to-lymphocyte ratio (NLR) are considered useful biomarkers of inflammaging and potential factors associated with mortality in the elderly population. However, the association of inflammaging with mortality among centenarians is less well known. We designed this prospective cohort study with long-term follow-up to explore the association of inflammaging with mortality among Chinese centenarians.

## Methods

This prospective cohort study was approved by the ethics committee of Hainan Hospital of Chinese People’s Liberation Army General Hospital. All participants provided written informed consent before participating in this study. The STROBE reporting guidelines were followed.

According to the household register provided by Hainan Civil Affairs Department, centenarians were surveyed for the China Hainan Centenarian Cohort Study from 2014 to 2016.^2^ Household surveys were performed to collect basic information with interview questionnaires, and blood tests were conducted following standard procedures by systematically trained doctors and nurses. Determination of mortality through 2021 was based on death registration records provided by China National Committee on Aging and Hainan Public Security Department. Cox regression analysis was used to determine associations of inflammatory biomarkers with mortality of centenarians. Nonlinear associations of CRP with mortality were analyzed by restricted cubic spline. *P* < .05 was considered statistically significant. The eAppendix in Supplement 1 provides additional details about the methods.

## Results

The 890 participants were a median (IQR) of 102 (101-104) years old, with 719 women (80.8%) and 786 individuals (88.3%) of Han ethnicity. Only 33 participants (3.7%) smoked cigarettes, 144 (16.2%) drank alcohol, and 124 (13.9%) drank tea. Mortality was associated with smoking cigarettes (hazard ratio [HR], 1.73; 95% CI, 1.11-2.69), drinking alcohol (HR, 1.30; 95% CI, 1.01-1.68), and NLR levels (HR, 1.05; 95% CI, 1.00-1.09) (Table). Restricted cubic spline analysis showed that there was a nonlinear association between CRP and mortality (Figure), with mortality increasing when CRP levels exceeded 16.74 nmol/L (to convert to milligrams per deciliter, divide by 95.2).

**Table.** Cox Regression Analysis With Mortality for Chinese Centenarians

Characteristics	Participants, No. (%) (N = 890)	HR (95% CI)	*P* value
Age, median (IQR), y	102 (101-104)	0.98 (0.95-1.02)	.35
Sex			
Male	171 (19.2)	1.03 (0.81-1.32)	.80
Female	719 (80.8)		
Ethnicity			
Han	786 (88.3)	0.93 (0.69-1.26)	.66
Li	93 (10.4)		
Smoking cigarettes	33 (3.7)	1.73 (1.11-2.69)	.02
Drinking alcohol	144 (16.2)	1.30 (1.01-1.68)	.04
Drinking tea	124 (13.9)	1.04 (0.80-1.34)	.79
C-reactive protein, median (IQR), nmol/L	0.19 (0.08-0.52)	1.00 (1.00-1.00)	.67
Neutrophil-to-lymphocyte ratio, median (IQR)	1.80 (1.34-2.51)	1.05 (1.00-1.09)	.04

Abbreviation: HR, hazard ratio.

SI conversion factor: To convert C-reactive protein to milligrams per deciliter, divide by 95.2.

**Figure.**
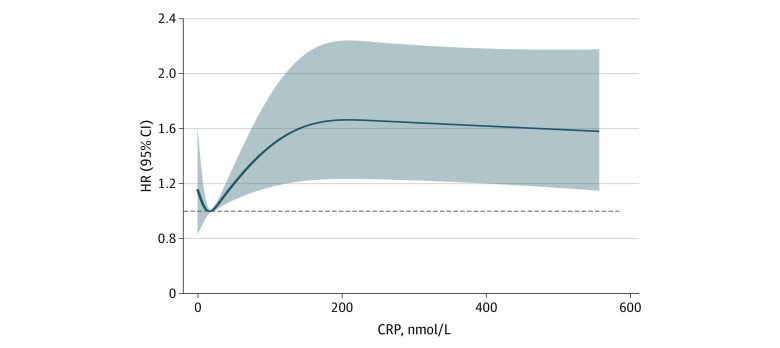
Restricted Cubic Spline Analysis of Association of C-Reactive Protein (CRP) Levels With Mortality Graph shows a nonlinear association of CRP with mortality (proportional hazards assumption, *P* > .05; nonlinear relationship test, *P* = .007; nonlinear *P* = .004). HR indicates hazard ratio. To convert CRP to milligrams per deciliter, divide by 95.2.

## Discussion

This cohort study of Chinese centenarians found a nonlinear association of mortality with CRP, a nonspecific inflammaging biomarker that is often used to evaluate inflammatory status of individuals in clinical practice.^3^ Elevated CRP levels have been associated with a higher risk of mortality.^4^ Inflammaging is one of the major causes of aging-related diseases that may affect aging phenotype and shorten the human lifespan.

NLR, an easily obtainable inflammaging biomarker, has also been recognized as a factor associated with risk of mortality.^5^ The Rotterdam Study^6^ demonstrated that NLR levels were independently associated with mortality in the elderly population. NLR levels were positively associated with mortality in centenarians in the current study.

Aging is often accompanied by a systemic chronic inflammatory state, with a weakened role of macrophages in recognizing and phagocytosing apoptotic neutrophils. Nicotine produced by smoking cigarettes activates neutrophils and releases factors that increase inflammatory reaction. Drinking alcohol affects macrophage composition, disrupts liver immunity, inhibits pathogen clearance, and exacerbates inflammatory damage. Both smoking and drinking were associated with increased mortality in this study. Extrapolation of our results to other populations is limited because of the influence of ethnicity and lifestyle.

This study found a linear association between NLR and mortality and a nonlinear association between CRP and mortality. Both CRP and NLR are easily obtainable and widely used and could be used as inflammaging biomarkers to monitor susceptibility to aging-related diseases among the elderly population.

## Notes

Supplement 1. **eAppendix.** Supplemental Materials and Methods

Supplement 2.
Data Sharing Statement
