# Supplementary material for: C-Reactive Protein, Neutrophil-to-Lymphocyte Ratio, and Long-Term Mortality in Chinese Centenarians
Source: JAMA Netw Open. 2023 Oct 30;6(10):e2340307. doi: 10.1001/jamanetworkopen.2023.40307 (PMC10616724; doi:10.1001/jamanetworkopen.2023.40307)
Supplement: Supplement 1. — eAppendix. Supplemental Materials and Methods [file jamanetwopen-e2340307-s001.pdf]

Supplemental Online Content

Zhu Q, Zhang Q, Chen C, et al. C-reactive protein, neutrophil-to-lymphocyte ratio, and long-term mortality in Chinese centenarians. *JAMA Netw Open*. 2023;6(10):e2340307. doi:10.1001/jamanetworkopen.2023.40307

eAppendix. Supplemental Materials and Methods

This supplemental material has been provided by the authors to give readers additional information about their work.

© 2023 Zhu Q et al. *JAMA Network Open.*

eAppendix. Supplemental Materials and Methods

Study population

This study was derived from China Hainan Centenarian Cohort Study (CHCCS)[6]. According to the household register provided by Hainan Civil Affairs Department, a full sample survey was conducted on centenarians in Hainan Province from 2014 to 2016. A total of 1002 centenarians were enrolled in this study, and 35 without blood samples and 77 with incomplete data were excluded. Finally, there were 890 centenarians included in this study. Until 2021, based on death registration records provided by China National Committee on Aging and Hainan Public Security Department, follow-up personnel determined death of centenarians and all other relevant information. This study was approved by the Ethics Committee of Hainan Hospital of Chinese People's Liberation Army General Hospital (Sanya, Hainan; Number: 301HNLL-2016-01). Written informed consent was obtained from each participant prior to the start of this study.

Standard procedures

The household survey method was used to collect basic information with interview questionnaires and blood tests conducted following standard procedure by systematically trained doctors and nurses who could communicate in local language. Age, gender and ethnicity are subject to participant's identity document card. Blood samples were routinely drawn by venipuncture and stored at 4°C, and transported to laboratory of our hospital within 4 hours. CRP was measured by an automatic biochemical analyzer (Cobas c702; Roche Diagnostics GmbH, Mannheim, Germany)

© 2023 Zhu Q et al. *JAMA Network Open.*

using Tina-quant C-Reactive Protein IV (CRP4). NLR was measured by an automated hematology analyzer (XN3000, Sysmex Corporation, Kakogama, Japan) using Fluorocell WDF and Lysercell WDF.

Statistical analyses

Medians and interquartile ranges are presented for continuous variables with skewed distribution, and counts and percentages are presented for categorical variables. Cox regression analysis was used to determine influence of inflammatory biomarkers on mortality of centenarians. Non-linear correlation between CRP and mortality was analyzed by restricted cubic spline (RCS). A P value <0.05 was considered statistically significant.

© 2023 Zhu Q et al. *JAMA Network Open.*
