# Supplementary material for: C-Reactive Protein, Neutrophil-to-Lymphocyte Ratio, and Long-Term Mortality in Chinese Centenarians
Source: JAMA Netw Open. 2023 Oct 30;6(10):e2340307. doi: 10.1001/jamanetworkopen.2023.40307 (PMC10616724; doi:10.1001/jamanetworkopen.2023.40307)
Supplement: Supplement 2. — Data Sharing Statement [file jamanetwopen-e2340307-s002.pdf]

Data Sharing Statement

Zhu. C-Reactive Protein, Neutrophil-to-Lymphocyte Ratio, and Long-Term Mortality in Chinese Centenarians. *JAMA Netw Open*. Published October 30, 2023.
doi:10.1001/jamanetworkopen.2023.40307

Data
Data available: No
